# Fine-tuning the evolutionary stability of recombinant herpesviral transmissible vaccines

**DOI:** 10.1098/rspb.2024.1827

**Published:** 2024-11-13

**Authors:** Baca Chan, Scott L. Nuismer, Hujaz Alqirbi, Jenna Nichols, Christopher H. Remien, Andrew J. Davison, Michael A. Jarvis, Alec J. Redwood

**Affiliations:** ^1^ Institute for Respiratory Health, University of Western Australia, Nedlands, WA 6009, Australia; ^2^ School of Biomedical Science, University of Western Australia, Nedlands, WA 6009, Australia; ^3^ Department of Biological Sciences, University of Idaho, Moscow, ID 83844, USA; ^4^ School of Biomedical Sciences, University of Plymouth, Plymouth PL4 8AA, UK; ^5^ MRC-University of Glasgow Centre for Virus Research, Glasgow G61 1QH, UK; ^6^ Department of Mathematics and Statistical Science, University of Idaho, Moscow, ID 83844, USA; ^7^ The Vaccine Group, Plymouth, Devon PL6 6BU, UK

**Keywords:** virus evolution, transmissible vaccines, vaccine safety, viral vaccine vector

## Abstract

Spillover of infectious diseases from wild animal populations constitutes a long-standing threat to human health for which few globally viable solutions have been developed. The use of oral baits laden with conventional vaccines distributed *en masse* represents one success story but is costly and practicable primarily for rabies risk reduction in North American and European carnivores. Efforts to expand vaccination to control pathogens within less accessible wildlife populations have raised interest in a new kind of vaccine capable of spreading pathogen-specific immunity through autonomous spread. However, such ‘transmissible’ vaccines raise concerns about the irrevocable release of genetically modified viruses into the environment. Herein, we explore the feasibility of an intrinsic strategy for transgene control within these vaccines based on the genetic destabilizing effect of *cis*-acting sequences flanking the heterologous transgene of interest. While suitable for the control of transgene stability within all types of DNA-viral vectored vaccines, this strategy has particular applicability to transmissible vaccines. Using a combination of experiments, mathematical modelling and whole-genome sequencing, we show that the rate of transgene loss can be controlled by varying the lengths of the direct repeat sequences. This opens a way for fine-tuning the lifespan of a transmissible vaccine in the wild.

## 1. Introduction

Recent estimates suggest that 60–75% of emerging infectious diseases enter the human population either directly from a reservoir animal species or via an intermediate animal host [1,2]. The SARS-CoV-2 pandemic is a salutary lesson of the societal impact of zoonotic pathogens following the acquisition of human-to-human transmission. SARS-CoV-2 infection has thus far killed over seven million individuals worldwide with an estimated cost of over 12 trillion US dollars [3,4] (https://data.who.int/dashboards/covid19/about). Outbreaks and epidemics of SARS, MERS, Ebola, H1N1 and Nipah virus infection further demonstrate the perils of relying on a reactive approach in managing the spillover of zoonotic pathogens, as do persistent spillovers of Lassa, rabies and Sin Nombre viruses [5,6].

One proven method is to prevent the spillover of zoonotic pathogens before they occur, by vaccination of the animal reservoir. Vaccines have been used effectively to prevent spillover of influenza A virus from chickens to humans [7] and rabies virus from carnivores (domestic dogs, red foxes and racoons) to humans [8–12]. Mass distribution of vaccine-laden baits has been used successfully to control or eliminate rabies from some wild animal species in the Americas and Europe [13–15], but this approach is typically limited to a small range of species and to high-income countries. An alternative approach that may significantly broaden the feasibility of wildlife vaccination involves the development of transmissible vaccines [16–20]. These vaccines can spread autonomously from animal to animal and thus offer a scalable approach for achieving high levels of immunological coverage through vaccination of reservoir species inhabiting remote areas where animals are difficult to access directly, where climatic conditions preclude bait-based approaches or where target animals are widely dispersed [21,22].

The use of recombinant vector designs instead of traditional approaches, such as attenuation, obviates many safety concerns associated with viral reversion [23–25]. Recombinant vector vaccines are live replicating viruses engineered to carry a foreign transgene, from which the expressed protein elicits immunity against the target pathogen. These vaccines have been shown to benefit from a self-adjuvanting effect generated by the viral vector, and their ability to replicate enables their self-dissemination by natural pathways [26,27]. As long as a recombinant vector vaccine is constructed from a benign, naturally circulating and species-specific virus, evolution is expected to favour the eventual elimination of the transgene and a return to the wild-type vector virus [18]. Even so, it may be desirable to incorporate a means of adjusting the rate of evolution and the longevity of the genetically modified virus in the environment [28]. For instance, controlling the lifespan of the transgene may reduce the spatial spread of the engineered virus and opportunities for recombination with other organisms, thereby addressing some regulatory concerns. Beyond transmissible vaccines, the ability to fine-tune the environmental longevity of genetically modified viruses also has implications in the development of immunocontraceptive vaccines for wildlife control [29,30] and virally vectored therapies [31].

Here, we study the feasibility of this approach using an engineered murine cytomegalovirus (MCMV). Cytomegaloviruses (CMVs) are appealing candidates for the development of self-transmissible vaccines owing to their strict host species specificity, their ability to induce robust immune responses, their large genomes capable of accommodating large inserts and their accessibility to established methods of genetic engineering [21,29]. Moreover, CMVs are benign in healthy hosts and are capable of superinfection, which is crucial for viruses such as CMV where infection is widespread [21,29,32]. To determine if we could control targeted transgene loss, we constructed a series of recombinant MCMVs containing a *LacZ* transgene flanked by direct repeats of varying lengths. Through extensive *in vitro* passaging and validation by whole-genome sequencing, we demonstrated the effective removal of the transgene via homologous recombination between flanking repeats of 250 base pair (bp in size. We fitted mathematical models to these data, which showed that both mutation and selection drive transgene loss. This vaccine recall strategy should be applicable to all DNA viral vectors that seek to incorporate a transgene decay capability in recombinant viruses.

## 2. Results

### (a) Strategy for cloning and constructing murine cytomegalovirus vectors expressing β-galactosidase

**Figure 1.**
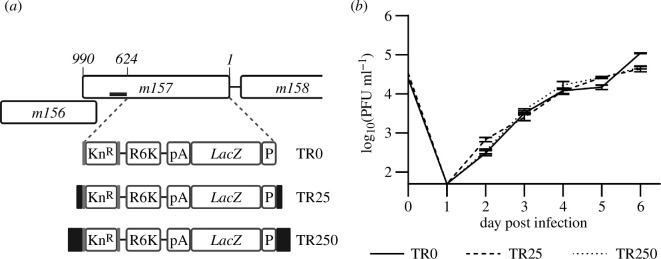
Murine cytomegalovirus (MCMV) vaccines containing a *LacZ* transgene flanked by repeat sequences. (*a*) The transgene contains a *LacZ* gene, an EF1α promoter (P), a bovine growth hormone (BGH) polyadenylation signal site (pA), a suicide origin of replication (R6K) and a kanamycin resistance cassette (Kn^R^) flanked by flippase recognition target (FRT) sites (grey lines). This was inserted into a truncated MCMV K181 strain (ARK14) resulting in a 624 bp deletion of the *m157* gene (nucleotides 1–624 deleted), denoted TR0. In additional constructs, this transgene was flanked by either 25 bp (TR25) or 250 bp (TR250) of *m157* repeat sequences (indicated by black line and black boxes). (*b*) Multi-step growth analyses (MOI 0.05) of MCMV-TR viruses in M2-10B4 cells demonstrated identical replication kinetics. The limit of detection is 50 PFU ml^−1^. Data are shown as mean ± s.d.

For this proof-of-principle study, a truncated MCMV genome lacking the *m07–m12* genes was chosen as the candidate DNA viral vaccine vector (ARK14 [33]). The deleted genes are dispensable for normal MCMV replication in cell culture [34]. The *LacZ* gene under the control of the EF1α promoter was chosen as the transgene to allow easy tracking of its loss by assaying for β-galactosidase (β-gal) activity. Along with the kanamycin resistance cassette, the transgene was inserted into the MCMV genome, at the same time deleting 624 bp of the *m157* gene, which is also non-essential for replication of MCMV in cell culture [35]. The transgene was flanked by direct repeats of either 0, 25 or 250 bp from *m157*, resulting in three constructs designated MCMV-TR0, MCMV-TR25 and MCMV-TR250 (figure 1*a*). We chose 25 bp as a minimal length sufficient for homologous recombination in mammalian cells [36]. A 10-fold longer sequence of 250 bp was additionally selected, as we reasoned that it would be sufficient to ensure homologous recombination for this proof-of-principle study of a self-recall transgene mechanism. No inadvertent mutations were detected by restriction fragment length polymorphism (RFLP) analyses (electronic supplementary material, figure S2C) or whole-genome sequencing of the bacterial artificial chromosome (BAC)-based constructs. All three viruses replicated with identical kinetics in M2-10B4 cells (figure 1*b*).

### (b) Rate of transgene expression loss is dependent on the length of direct repeat sequences

**Figure 2.**
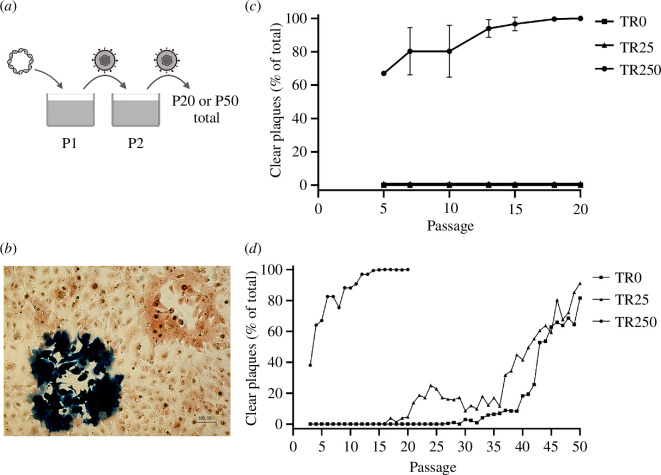
Passaging of MCMV-TR viruses in tissue culture. (*a*) Infectious virus was resuscitated following transfection of MCMV-BAC DNA into M2-10B4 cells. This is denoted as passage 1 (P1). In total, 20% of the virus-containing supernatant was then transferred onto naive cells, denoted P2. Every 72–96 h, this process was repeated as indicated for a total of 20 or 50 passages. The supernatants at indicated passages were subjected to standard plaque assay coupled with 5-bromo-4-chloro-3-indolyl-β-D-galactopyranoside (X-gal) staining and neutral red counterstaining. (*b*) Representative microscopic image of M2-10B4 cells infected with MCMV-TR0. Blue plaque indicates β-galactosidase (β-gal) expression, whereas clear plaques originate from virus that has lost β-gal expression. Scale bar = 100 µm. (*c*) Loss of transgene expression over 20 passages performed in triplicates. Rate of loss is shown as the percentage of clear plaques relative to total plaques. Data are shown as mean ± s.d. (*d*) Loss of transgene expression over 50 passages. For all passaging, a minimum of 100 plaques were counted for each data point.

There are multiple mechanisms by which β-gal expression could be lost, including loss of protein production owing to nonsense mutations, deletions within the *LacZ* gene and mRNA degradation. Here, we are interested in the controlled excision of the *LacZ* gene, rather than merely silencing of protein function. To determine whether the *LacZ* transgene is excised from MCMV vector genomes via homologous recombination upon passaging in cell culture and whether the length of the direct repeat sequences affects the rate of excision, the three MCMV-TR viruses were passaged 20 times in M2-10B4 cells (figure 2*a*). Loss of transgene expression was tracked via 5-bromo-4-chloro-3-indolyl-β-d-galactopyranoside (X-gal) staining, which enables the differentiation of plaques expressing β-gal (blue plaques) or not expressing β-gal (clear plaques; figure 2*b*). Upon serial passaging, β-gal expression was lost rapidly from MCMV-TR250 (figure 2*c*). At passage 5 (P5), which was the earliest passage at which plaques were monitored, loss of β-gal expression was detected in >60% of the population. By P13, >90% of the population no longer expressed β-gal, and by P20, no β-gal-expressing plaques were detected. In contrast, the transgene remained stable for the entire course of 20 passages in MCMV-TR0 and MCMV-TR25.

To determine whether *LacZ* expression is eventually lost from MCMV-TR25 and MCMV-TR0, the experiment was repeated, extending the number of passages to 50 and monitoring β-gal expression at each passage from P3 (figure 2*d*). Confirming our previous observations, β-gal expression was lost rapidly in MCMV-TR250, with >90% loss by P11 and >99% by P14. Passaging of MCMV-TR250 was terminated at P20 when only β-gal-negative plaques were evident. In contrast, β-gal-negative plaques were first observed in MCMV-TR25 at P16. The proportion of clear plaques continued to rise until P24 and then decreased until P36, reaching 90% loss by P50. MCMV-TR0 remained stable until P26, after which β-gal-negative plaques accumulated to 80% by P50. Combined, these results suggest that the kinetic loss of β-gal activity was linked to the length of the repeat sequences flanking the *LacZ* transgene.

### (c) Both mutation and selection drive transgene expression loss

**Figure 3.**
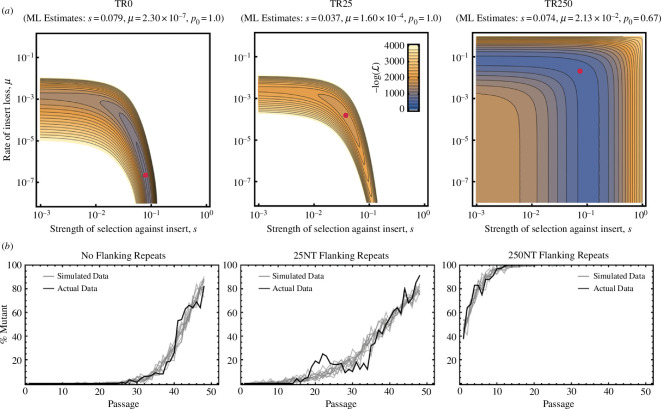
Transgene expression loss is driven by both mutation and selection. (*a*) The negative log-likelihood of the serial passage data as a function of the mutation rate, *μ*, and the strength of selection, s, for flanking repeats of three different lengths. The red dot indicates the maximum likelihood (ML) estimate for each panel and the colour shading the value of the negative log-likelihood. Although we also estimated the parameter $p_{0}$, defining the initial frequency of virus carrying the transgene, its value was fixed at its maximum likelihood value in each panel for simplicity. White areas of the figure are those with negative log-likelihoods greater than the values shown on the scale bar. (*b*) Comparisons of simulated (grey lines) and real (black line) serial passage data for designs with different length flanking repeats. Simulated serial passage data depict the results of ten independent simulations where the observed frequency of mutant virus at each time point was calculated from a random sample of 100 plaques with the probability of sampling intact virus carrying the transgene given by equation (4.2) with parameters *μ*, s and $p_{0}$ set to their maximum likelihood estimates. NT, nucleotide.

We next sought to disentangle the relative contributions of mutation and natural selection to the loss of β-gal activity by fitting simple mathematical models to the passage data. We used our previously developed method [37], which models the process of transgene loss driven by spontaneous mutation and natural selection to estimate model parameters using maximum likelihood. This framework assumes that mutation removes the entire transgene in a single step at rate *μ*, and that carrying the transgene reduces the rate of viral replication by an amount *s*. Our maximum likelihood estimates demonstrated that the increased rate of β-gal decay, observed with longer flanking repeats, likely stemmed from increases in the rate of spontaneous transgene loss through mutation. Specifically, we estimated values for the mutation rate, *μ*, of $2.30\times 10^{-7}$, $1.60\times 10^{-4}$ and $2.13\times 10^{-2}$, respectively, for MCMV-TR0, MCMV-TR25 and MCMV-TR250. Estimates for the initial frequency of virus carrying the transgene were consistent with these mutation rates, with MCMV-TR0 and MCMV-TR25 cultures estimated to be pure at the start of serial passaging ($p_{0}=1.00$), but the MCMV-TR250 culture was estimated to already contain a substantial frequency of virus lacking the insert at the start of passaging ($p_{0}=0.67$). The extensive infiltration of virus lacking the transgene at the start of serial passaging for MCMV-TR250 was likely a consequence of the extremely high mutation rate in this design. In contrast to mutation, our estimates for the strength of selection are relatively insensitive to the length of flanking repeats, demonstrating robust selection against the *LacZ* transgene in all cases (0.079, 0.037 and 0.074, respectively). The likelihood surfaces for each case, along with the maximum likelihood estimates, are shown in figure 3*a*.

Simulating data using our fitted models and stochastic binomial sampling demonstrated excellent fits for MCMV-TR0 and MCMV-TR250. In contrast, the fit to data from MCMV-TR25 was less satisfactory, with a large discrepancy between predicted and observed values occurring between P18 and P34 (figure 3*b*). The discrepancy between real and simulated data arose because of a non-monotonic pattern of increase observed for the frequency of β-gal-negative virus. We hypothesized that this pattern resulted from clonal interference between an initial mutant virus that eliminated β-gal function and a secondary mutant virus that also eliminated β-gal function but that yielded a greater increase in viral fitness. This hypothesis could explain why our estimated value of selection for MCMV-TR25 was only half that estimated for MCMV-TR0 and MCMV-TR250 (see below).

### (d) Sequencing reveals transgene excision can occur via non-homologous recombination

**Figure 4.**
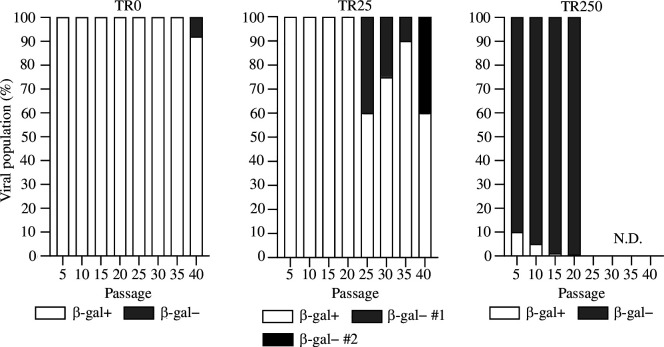
Size of direct repeats dictates transgene loss by homologous or non-homologous recombination. Viral genomes at every fifth passage to P20 for TR250 and to P40 for TR0 and TR25 were subjected to whole-genome sequencing. The *LacZ* transgene flanked by 250 bp of repeat sequences is cleanly excised via homologous recombination between flanking repeats and already detected in 90% of the viral population by P5. In the absence of suitably large direct repeat sequences, the *LacZ* transgene is only lost following extensive *in vitro* passaging (first detected at P40 for TR0 and at P25 for TR25) and via non-homologous recombination leading to large genome deletions. N.D., not detected; β-gal, β-galactosidase.

To investigate whether β-gal-negative plaques arose during passage as a result of transgene excision involving homologous recombination between direct repeats in MCMV-TR250 and MCMV-TR25, viral cultures from figure 2*d* were subjected to whole-genome sequencing at every fifth passage (figure 4 and electronic supplementary material, table S1). MCMV-TR250 cultures up to P20 and MCMV-TR0 and MCMV-TR25 cultures up to P40 were analysed. There was excellent concordance between sequence and plaque assay data, indicating that the lack of β-gal activity was entirely driven by *LacZ* transgene excision rather than functional inactivation. Importantly, sequencing confirmed that transgene loss in MCMV-TR250 was via homologous recombination between the flanking repeat sequences, leading to precise excision of the transgene and reversion to the parental sequence. The transgene in MCMV-TR0 remained stable until P35 but had been lost from approximately 8% of the population by P40. This non-homologous recombination-mediated excision resulted in the deletion of MCMV sequences 510 bp upstream and 890 bp downstream of the transgene.

Modelling suggested that loss of transgene expression in MCMV-TR25 occurred via a two-step process, and this was confirmed by the sequencing data. At P25, the transgene had been lost from 40% of genomes via non-homologous recombination. Loss of the transgene was accompanied by the deletion of MCMV sequences from 7397 bp upstream and 134 bp downstream from the transgene. An additional mutational event was detected at P35, involving a second recombinant making up 40% of the viral population by P40. This recombinant apparently outcompeted the original recombinant, which was undetectable at P40. As with the original deletion, this deletion also occurred via non-homologous recombination, resulting in 2283 bp partial loss of the transgene as well as 5514 bp beyond.

The sequencing data also revealed the occurrence of multiple mutations elsewhere in the genomes (electronic supplementary material, table S1), including those in genes MCK-2 and Sgg-1. These data are consistent with the events that occur routinely during prolonged passaging of MCMV in cell culture and that have been reported elsewhere [38,39].

### (e) Modelling the effect of differing levels of fitness cost on transgene loss

Because the strong selection we estimated against *LacZ* may be significantly greater than that acting against other transgenes used to develop recombinant vector viruses, we calculated the expected half-life for transgenes with a range of fitness costs. Specifically, we solved for the time required for the frequency of the transgene to decrease from 100% $\left(p=1\right)$ to 50% (*p* = 0.5) within the viral population:


(2.1) $$\tau_{50}=-\frac{ln\left(\frac{\mu (1-s)}{s+2\mu (1-s)}\right)}{r(s+\mu (1-s))}.$$

This simple analysis predicted that transgene loss may proceed extremely slowly in the absence of strong selection, with even a weakly selected transgene flanked by a 25 bp repeat having a half-life of almost 2 years (electronic supplementary material, figure S1). Thus, although our results support the basic principle of using flanking repeats to adjust the half-life of genetically engineered viruses, they also demonstrate that fine-tuning the half-life to meet specific objectives will require intimate knowledge of selection acting on any specific transgene.

## 3. Discussion

Transmissible vaccines have been proposed as a novel solution to combating zoonotic infections within their reservoir hosts, thereby preventing spillover events that pose significant risks to livestock and human populations [18]. A self-propagating vaccine delivered to difficult-to-access wildlife populations has many advantages, but real-world use could be impeded by safety concerns over the release of a genetically modified virus that might circulate in perpetuity [23]. Our study is the first demonstration that repeat sequences flanking a transgene could be used as a safety mechanism to accelerate the reversion of a vaccine vector virus to the original wild-type virus. Specifically, we showed that this strategy instituted in an MCMV vaccine vector leads to homologous recombination-mediated excision of the transgene. Furthermore, the rate of transgene loss via homologous recombination is defined by the size of the flanking repeat sequences. Genome sequencing demonstrated that the flanking repeat sequences not only mediate excision but are necessary for scarless excision of the transgene. In the absence of suitable repeats of adequate length, excision is instead mediated by non-homologous recombination events, resulting in large, unpredictable deletions of the transgene and surrounding sequences.

Although the results demonstrated transgene excision for all lengths of flanking repeats tested (0, 25 and 250 bp), the evolutionary forces driving excision and the mechanisms involved in excision varied. When the transgene was flanked by 250 bp repeats, excision was rapid, and our maximum likelihood estimates suggested that this could be attributed to a high mutation rate. Indeed, maximum likelihood estimates suggested that flanking repeats of this length render the transgene so unstable that a substantial proportion of virus had lost the transgene during generation from the BAC and before serial passaging began. In contrast, transgene excision was much slower in the absence of flanking repeats, and our maximum likelihood estimates suggest this is attributable to a much lower rate of mutation. Finally, when the direct repeats were 25 bp in length, the rate of transgene excision was intermediate, and the maximum likelihood estimates suggested a reduced strength of selection. However, since the transgene was identical in all three cases, there was no *a priori* reason to anticipate a reduced strength of selection in this case. When coupled with the distinct lack of fit between the modelling predictions of our model and the temporal pattern of transgene loss that was observed (figure 3*b*), the data led us to suspect the involvement of a two-step process in excision in which an initial mutation arose but failed to proliferate and was soon outcompeted by a more favourable second mutation. This prediction was borne out by genome sequencing. Therefore, direct repeat sequences allow for restoration of wild-type viral sequences when of sufficient length to promote homologous recombination. However, when repeat sequences are not provided or are of insufficient length, then loss of transgenes can occur via an unpredictable, sometimes multi-step process, which fails to restore wild-type viral genomes.

This study constitutes a proof-of-concept approach to developing better vaccine safety mechanisms. Its conclusions are borne out of studies conducted in cell culture, and further studies in the animal host are necessary to understand their applicability in the face of host immune responses. An intact immune response will add complexity to the pressures exerted on the transgene, but the ability to elicit an immune response is also crucial to the efficacy of the vaccine. In practice, a key balance must be struck, wherein the transgene is present in the population for long enough to elicit protective immunity in a large percentage of the population. However, it is likely that immune factors will drive the loss of the transgene, and therefore the length of the flanking repeats will need to be adjusted accordingly. Here we chose two repeat sequences expected to represent the lower and upper lengths required for homologous recombination. In future studies, we aim to use smaller incremental variations in flanking sequence sizes to more finely tune our transgene recall strategy. Parenthetically, the transgene flanked by 250 bp repeats was rapidly deleted in cell culture, thus highlighting the difficulties that may be associated with generating large high-titre stocks of vaccine vector when the repeats are relatively long.

The *LacZ* transgene provided us with an easily traceable marker, for which we could rapidly detect and quantify loss at each passage. There remain, however, many factors to consider for a recombinant vaccine with real-world implications. Our insert of nearly 6 kb, including the 3 kb *LacZ* gene, is relatively large and, as shown from our modelling (figure 3*a*), under strong selective pressure even in the absence of host immunity. The size of the MCMV genome is relatively conserved [40], suggesting packaging constraints to genome size. Therefore, a smaller antigen construct would likely be used in future recombinant vaccine studies. Such studies would allow us to model different selection pressures on the vaccine, for example, the role that DNA packaging constraints play on transgene loss.

For these *in vitro*-based studies, we chose to place our transgene partially over the *m157* gene, an immune evasion gene dispensable for MCMV replication in cell culture [35]. The *m157* gene is a member of the *m145* gene family; many of these genes encode immune evasion proteins and are not required for MCMV replication in cell culture [41]. Owing to the redundant nature of these genes in cell culture, non-homologous recombination-mediated excision of the transgene coincided with large deletions of neighbouring sequences. While this was well tolerated by MCMV in cell culture, viruses with such deletions would be severely attenuated and subsequently selected against in the host. Hence, in designing recombinant vaccines for future *in vivo* studies, insertion sites and flanking sequences for homologous recombination will need to be selected to ensure scarless transgene loss.

The transgene in this study, *LacZ*, is not a pathogen-related antigen, and therefore the recombinant MCMVs that were constructed are not vaccines. We next seek to explore the applicability of our approach in host animals by targeting a suitable zoonotic pathogen. An important step in this work was our recent identification and full characterization of multiple CMVs in *Mastomys natalensis*, the reservoir species for a range of zoonotic pathogens, including Lassa virus [42]. Although we have focused on transmissible vaccines as an application, our work suggests a general mechanism through which the environmental longevity of engineered viruses can be fine-tuned.

## 4. Material and methods

### (a) Construction of murine cytomegalovirus containing a *LacZ* transgene flanked by repeat sequences

The vaccine vector for this study is a truncated K181 strain of MCMV lacking genes *m07–m12* (ARK14 [33]. The construction design is outlined in electronic supplementary material, figure S2. First, 25 and 250 bp of MCMV repeat sequences corresponding to the *m157* gene (nt 219 338–nt 219 362 and nt 219 113–nt 219 362, respectively, accession no. PP756678) flanked by *Eco*RI sites were synthesized by GeneArt (Invitrogen), named TR plasmids. These fragments were inserted into the *Eco*RI site of pEF1a/EBOV/Kn to give pEF1a/EBOV/Kn/TR. The *LacZ* gene was amplified from pEQ176 (Addgene, no. 83943) and inserted into the *Nhe*I/*Not*I sites, replacing EBOV-GP. The resultant plasmid, pEF1a/LacZ/Kn/TR, was linearized by *Pme*I digestion and inserted into the ARK14 BAC by standard ET recombination as previously described [33]. Insertion of the transgene into the MCMV genome led to deletion of 624 bp from the start of the 990 bp *m157* gene and was flanked by either 0, 25 or 250 bp MCMV repeat sequences (denoted MCMV-TR0, MCMV-TR25 and MCMV-TR250,).

### (b) Rescue of infectious virus

MCMV BAC DNA was isolated using the NucleoBond Xtra Midi kit (Macherey-Nagel) and transfected into the murine bone marrow stromal M2-10B4 cell line by calcium phosphate transfection using the CAPHOS transfection kit (Merck) according to manufacturer’s instructions. Cells were monitored for plaque development, and once 100% cytopathic effect in the monolayer was observed, virus from the supernatant and cells was collected and used to infect fresh uninfected M2-10B4 cells. This initial infectious virus harvest from MCMV BAC DNA transfected M2-10B4 cells was deemed passage 1 (P1).

### (c) Murine cytomegalovirus multi-step growth curves

M2-10B4 cells were infected in triplicate at a multiplicity of infection (MOI) of 0.05 for 1 h at 37°C followed by a citric acid buffer (pH 3.0) wash to remove unbound virus. Fresh medium was added to the culture to initiate the start of the time course. In total, 100 μl of the supernatant (representing 2.5% of the total supernatant) was harvested and replaced with fresh medium every 24 h for 6 days and stored at −80°C for titration by standard plaque assay.

### (d) Passaging in cell culture

The day before infection, 1 × 10^5^ M2-10B4 cells were seeded per well of a 24-well tray. One hundred microlitres of MCMV-containing supernatant (equivalent to 1 × 10^2 ^– 1 × 10^4^ plaque-forming units, PFU) was mixed with 400 μl of cell culture medium and added directly to cells. Once 100% cytopathic effect (CPE) was observed (72–96 h post-infection), 100 μl of the supernatant was collected and again mixed with 400 μl of fresh cell culture medium and added to uninfected cells. Every round of infection of fresh uninfected cells was counted as a new passage. Supernatants were subjected to standard plaque assay and X-gal staining to determine β-gal expression.

### (e) X-gal staining and neutral red counterstaining of murine cytomegalovirus-infected cells

Virus was quantified using a standard plaque assay. At 5 days post-infection, 300 μl of 0.5% glutaraldehyde solution was added to the cells without the removal of carboxymethyl cellulose, incubated for 20 min and washed with distilled water followed by two rounds of washing with phosphate-buffered saline (PBS). A third PBS wash was left in place for 10 min. One hundred and fifty microlitres of X-gal stain solution (2 mM MgCl_2_, 5 mM C_6_N_6_FeK_3_, 5 mM C_6_FeK_4_N_6_, 0.01% w/v Triton X-100, 0.4 mg ml^−1^ X-gal, PBS) was added per well and left to incubate for 30 min in the dark. To counterstain, the X-gal stain was washed off with distilled water, and 150 μl of neutral red solution was added to the cells and left for 15 min before washing off with water. Plaques originating from the virus expressing β-gal stained blue, whereas the absence of β-gal activity resulted in clear plaques. A minimum of 100 plaques were counted per virus per time point.

### (f) Whole-genome sequencing

DNA was extracted from 1 × 10^6^ MCMV-infected M2-10B4 cells using a DNeasy Blood and Tissue kit (Qiagen). Samples from every fifth passage up to P20 for MCMV-TR250 and P40 for MCMV-TR0 and MCMV-TR25 were sequenced. DNA was sheared to an approximate size of 450 bp using a Covaris LE220 sonicator. Sequencing libraries were then prepared up to the adapter ligation stage using a Kapa Biosystems LTP library preparation kit for Illumina platforms. At that point, the samples were indexed using NEBNext Multiplex Oligos for Illumina 96 unique dual index primer pairs sets 1–4 (New England Biolabs) with seven PCR cycles. Sequencing was performed on an Illumina Miseq instrument using a MiSeq v2 300 cycle kit to yield paired-end reads of 151 nt.

### (g) Analysis of sequence data

Sequence reads were trimmed using Trim Galore Sequence v. 0.6.6 (https://www.bioinformatics.babraham.ac.uk/projects/trim_galore/) and then aligned to the relevant BAC sequence using Bowtie 2 v. 2.4.2 [43] and Samtools v. 1.12 [44]. Alignments were inspected visually using Tablet v. 1.21.02.08 [45]. Proportions of mutant populations were estimated by counting trimmed reads containing the parental or mutated sequences using the grep command-line utility instituted in Unix. Detailed results are provided in electronic supplementary material, table S1: mutations detected in the profiles sheet and read-counting data in the proportions sheet.

### (h) Modelling mutation and selection

We disentangled the relative contributions of mutation and natural selection to transgene loss by fitting simple mathematical models to the passage data using maximum likelihood. Specifically, we used our method reported previously [37], which models the process of transgene loss driven by spontaneous mutation and natural selection against transgene carriage. This framework assumes that mutation removes the entire transgene in a single step at rate μ, and that carrying the transgene reduces the rate of viral replication by an amount *s*. As long as viral populations are transferred during the exponential growth phase (e.g. well before carrying capacity is reached), the change in the population sizes of engineered and revertant virus lacking the transgene is given by


(4.1*a*) $$\frac{dN_{E}}{dt}=rN_{E}(1-s)(1-\mu ),$$

(4.1*b*) $$\frac{dN_{R}}{dt}=rN_{R}+rN_{E}\left(1-s\right)\mu ,$$

where $N_{E}$ and $N_{R}$ are the population sizes of engineered and revertant viruses, respectively, r is the baseline growth rate of virus in culture, μ is the rate of spontaneous transgene loss through mutation/recombination and s is the strength of selection caused by carrying the transgene. As long as population sizes of virus remain sufficiently large for stochastic effects to be ignored, these expressions can be used to determine the frequency of virus carrying the transgene over time [37]:


(4.2) $$p\left(t\right)=\frac{p_{0}\left(s\left(1-\mu \right)+\mu \right)\;exp\left(-rt\left(s\left(1-\mu \right)+\mu \right)\right)}{\mu +s\left(1-\mu -p_{0}\left(1-exp\left(-rt\left(s\left(1-\mu \right)+\mu \right)\right)\right)\right)},$$

where $p_{0}$ is the frequency of virus carrying the transgene at the start of the experiment $\left(t=0\right)$.

Before we can estimate the parameters of interest in (4.2), we must estimate the value of the nuisance parameter, r. This parameter quantifies the intrinsic rate of increase for the virus in the absence of the transgene. To estimate r, we fit a standard model of exponential growth to the multi-step growth curve for parental ARK14 reported previously [33] using maximum likelihood. Specifically, we assume viral growth is exponential and that viral population size observed at each time point is drawn from a Poisson distribution with rate parameter equal to the expected population size at that time. This leads to the likelihood function


(4.3) $$L=\prod_{t=1}^{\tau }\frac{exp\left(-N\right)N^{X_{t}}}{X_{t}!},$$

where $N=N_{0}exp\left(rt\right)$ and $X_{t}$ are the observed viral population size at time *t*. We maximized the log-likelihood of 4 to yield estimates for the initial viral population size $\left(N_{0}=153.4\right)$, and intrinsic rate of increase (r=0.045). We used this estimated value of r in all downstream calculations.

Although results presented previously [37] focus on joint estimation of only mutation and selection, here we also estimate the initial frequency of virus carrying the transgene. Estimating the initial frequency of virus-carrying transgene is more flexible and allows us to study cases where revertant virus was present at the start of the experiment. We used the likelihood function defined previously [37] to estimate parameter values for each of the three datasets (TR0, TR25 and TR250), with maximization of the log-likelihood performed using the NMaximize function in Mathematica. After parameter estimation, the model was repeatedly simulated using the maximum likelihood parameter estimates to check for lack of fit. These simulations used equation (4.2) to predict the frequency of the transgene at each time point and created simulated data by drawing 100 random samples at each time point to mimic the sampling in the experiment, which relied on assaying 100 plaques.

## Data Availability

The complete sequences of the parental BAC and the derivative BACs containing the *LacZ* transgene have been deposited in NCBI GenBank under accession numbers PP756678, PP756681, PP756680 and PP756679. Supplementary material is available online [46].
